# Magnetic Metal Oxide-Based Photocatalysts with Integrated Silver for Water Treatment

**DOI:** 10.3390/ma15134629

**Published:** 2022-07-01

**Authors:** George V. Belessiotis, Pinelopi P. Falara, Islam Ibrahim, Athanassios G. Kontos

**Affiliations:** 1 National Center for Scientific Research “Demokritos”, Institute of Nanoscience and Nanotechnology, 15310 Athens, Greece; g.belessiotis@inn.demokritos.gr (G.V.B.); i.ibrahim@inn.demokritos.gr (I.I.); 2 School of Chemical Engineering, National Technical University of Athens, 15780 Athens, Greece; pin.falara@gmail.com; 3 Department of Chemistry, Faculty of Science, Al-Azhar University, Cairo 11884, Egypt; 4 Department of Physics, School of Applied Mathematical and Physical Sciences, National Technical University of Athens, 15780 Athens, Greece

**Keywords:** silver (Ag), magnetic composite, photocatalysis, pollutant degradation, ferrite, synthesis method

## Abstract

In this review, the most recent advances in the field of magnetic composite photocatalysts with integrated plasmonic silver (Ag) is presented, with an overview of their synthesis techniques, properties and photocatalytic pollutant removal applications. Magnetic attributes combined with plasmonic properties in these composites result in enhancements for light absorption, charge-pair generation-separation-transfer and photocatalytic efficiency with the additional advantage of their facile magnetic separation from water solutions after treatment, neutralizing the issue of silver’s inherent toxicity. A detailed overview of the currently utilized synthesis methods and techniques for the preparation of magnetic silver-integrated composites is presented. Furthermore, an extended critical review of the most recent pollutant removal applications of these composites via green photocatalysis technology is presented. From this survey, the potential of magnetic composites integrated with plasmonic metals is highlighted for light-induced water treatment and purification. Highlights: (1) Perspective of magnetic properties combined with plasmon metal attributes; (2) Overview of recent methods for magnetic silver-integrated composite synthesis; (3) Critical view of recent applications for photocatalytic pollutant removal.

## 1. Introduction

One of the great dangers facing humanity is the depletion of natural resources. For water specifically, the problem of shortage is all but gone. In 1960, only 9% of the world’s population was facing chronic water shortage issues [1], while now nearly 50% of the global population has to manage moderate shortage, with around 10% of that population facing almost complete lack of water [2,3]. Many measures have been employed to balance this ever-increasing threat, such as irrigated areas, groundwater utilization and reservoir storage [1], as this increase in water shortage does not only affect agriculture but human health itself as well and can even increase risks of diseases [2]. The need for low-cost water treatment is especially evident in countries such as Pakistan, where the increased dependency on severely threatening contaminated water (by ~80% of the population) [4] necessitates methods such as low-cost water filters for increased availability of affordable drinking water throughout the country. 

In recent times, the environmental impact of the technologies employed for basic functions concerning water/air quality control or energy, along with their dependence on finite resources, has become a significant issue, increasing the interest in green technologies power by light [5,6,7]. One such green and affordable technology for water purification is photocatalysis, utilizing light-activated semiconductors (SCs). In many applications, there is great interest in composite materials that offer the combined advantages of their respective components [8,9,10], with photocatalytic composites being especially effective in the removal of pollutants from aquatic solutions. There are several requirements in order to prepare a good composite photocatalyst. Among the desired attributes is a wider wavelength-range light response (UV and visible) and, ideally, a good response under solar light. Another significant need is the ease of removal of the photocatalytic material from the treated water solution, as often the photocatalyst itself can cause issues for the water quality. Silver-enhanced magnetic materials, which can fulfil these requirements, have seen a surge in popularity. Magnetic materials are a common type of photocatalyst, from iron oxides to the spinel ferrite family (MFe_2_O_4_, where M is a divalent metal cation), whose magnetic properties offer several enhancements when used alone or as part of a composite photocatalyst, with a primary advantage being their easy removal from a solution through magnetic means (such as a simple magnet). On the other hand, silver (Ag) nanoparticles, having a low cost [11] and offering plasmonic-based enhancements [11] and antibacterial properties [12] are a great fit as components in photocatalytic composites for water purification under a wide irradiation wavelength range. Thus, a magnetic/silver composite combines the advantages of magnetic properties with those of plasmonic nanoparticles, and these composites are able to perform well under UV and visible light, are effective against pollutants and pathogenic bacteria and can be easily separated from a solution with a simple magnet [13,14].

In this work, the latest developments in the synthesis and photocatalytic pollutant degradation applications of magnetic silver-integrated composites are presented. In the Section 2, the basics of photocatalysis and magnetic/silver composites are reviewed. In the Section 3, a detailed survey of the latest materials and their synthesis techniques is provided. In the Section 4, a critical analysis of the latest developments in the photocatalytic application of such composites for pollutant removal is presented. Finally, in the Section 5 a perspective on future research based on our survey is offered.

## 2. Magnetic Ag-Integrated Photocatalysts

### 2.1. Photocatalysis for Pollutant Removal

As a byproduct of the industrial revolution, numerous types of pollutants, from organic compounds to pathogenic microbes, have risen to threaten humans and the general environment and, in response, numerous methods have been designed and employed for polluted water treatment. Concerning organic pollutants, the employment of dyes and water by textile and plastic industries for coloring purposes, leads to harmful dye-based pollutants in wastewater with adverse effects on the environment [15]. Many techniques used for the removal of organic compounds often require additional treatment of byproducts. There is also the issue of non-organic pollutants: one of the more infamous pollutants, hexavalent chromium (Cr^6+^) [16], originating from the waste products of the chrome electroplating industry, presents carcinogenic properties that make it an extremely dangerous water pollutant. The high cost of Cr^6+^ removal techniques, such as coagulation or reverse osmosis, usually restricts them to large-scale utilization. With harmful waste from industrial sources, that do not naturally degrade, and chemicals from agricultural/pharmaceutical products finding their way into the environment, the need for a sustainable low-cost method for pollutant removal is becoming increasingly more urgent. An environmentally friendly method that can target a variety of pollutant types is photocatalysis [17]. 

**Figure 1:**
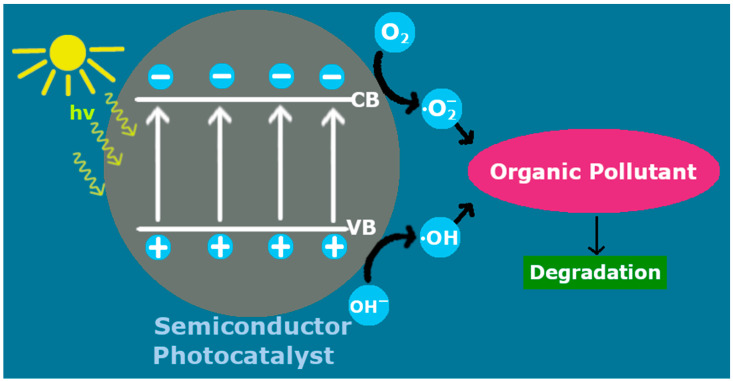
Photocatalytic treatment of organic pollutants by an irradiated photocatalyst.

In a typical photocatalytic process, after a semiconductor is irradiated with photons of higher energy than its band gap, electron/hole pairs are generated in its conduction/valence bands. These charged pairs are able to reduce/oxidize adjacent molecules, provided that the energy bands of the photocatalyst are properly positioned relative to the reactant’s redox levels [17]. However, a possible recombination of the electrons/holes can impair this activity. The photoexcited electrons can reduce adsorbed O_2_ into superoxide radicals (**·**O_2_), while the reaction of H_2_O with holes leads to hydroxyl radicals (**·**OH) [18]. These radicals, in turn, can function as active species for the decomposition of a pollutant (Figure 1). For example, the hydroxyl radicals can oxidize organic compounds into small and much less toxic molecules [19]. The application of photocatalysis extends even further than organic pollutant degradation: photocatalytic chromium treatment can be an affordable, green and efficient technique for the neutralization of this dangerous pollutant; Cr^6+^ can be reduced to its trivalent variation, Cr^3+^, which presents severely lower toxicity, via a photocatalytic reduction reaction [16,20,21,22]. 

The most common photocatalytic materials are metal oxides [18]. The photon energy of the irradiation must exceed the band gap of the catalyst for proper absorption and charge separation and TiO_2_, the most well-known photocatalyst, with its 3.2 eV band gap, absorbs a negligent portion of visible light, thus it is only suitable for photocatalytic operation under UV light. However, a photocatalyst should be active under both UV and visible light [18,23]. This is the case with magnetic hematite (α-Fe_2_O_3_) [24,25], which has significant absorption in the sunlight spectrum (around 40%) due to its smaller band gap [18]. Visible-range light absorption can be expected from several magnetic materials (e.g., the MFe_2_O_4_ family) [20,26].

### 2.2. Magnetic Materials and Silver Enhancement 

Among the most common magnetic materials used in photocatalysis are iron oxides having appropriate valence energy levels and narrow band gaps together with corrosion resistance and stability under irradiation. Besides FeO (iron (II) oxide), the most interesting forms in which iron oxides can be obtained through the usual synthesis methods are α/γ-Fe_2_O_3_ (iron (III) oxide phases: hematite/maghemite) and Fe_3_O_4_ (magnetite = Fe(II)Fe(III)_2_O_4_), with differences in their saturation magnetization and other attributes [27] (Hematite is anti-ferromagnetic material with small bulk magnetic susceptibility, while magnetite and maghemite are ferrimagnetic with large bulk magnetic susceptibility [28]). In general, ferrites (ferrimagnetic materials with great stability and tolerance to even severe basic/acidic conditions) are very promising for water treatment applications [20,29]. Though there are different types of ferrite structure, the category of ferrites that holds the most significance are the semiconducting spinel ferrites (also known as ferrospinels): cubic MFe_2_O_4_ structures, with M being a divalent cation such as cobalt, zinc, magnesium, etc. [30] or combinations of them [31]. They are characterized by high saturation magnetization and increased permeability among other interesting properties, resulting in an upsurge of related research in recent years [28]. For spinel ferrite magnetic nanostructures specifically, known advantages include chemical stability, mechanical hardness and high magnetic coercivity [32]. In general, the aggregation state of magnetic nanoparticles can affect their magnetic behavior and ferrospinel nanoparticles, in particular, usually exhibit superparamagnetic behavior (with an absence of remnant magnetization), while in cluster form they can exhibit ferrimagnetic behavior [33].

Magnetic photocatalysts, in general, have attracted significant interest due to the effects of intrinsic and external magnetic fields on them and the enhancements that their manipulation can offer to photocatalytic water purification applications. It is known that external magnetic field application during photocatalytic reactions can enhance e^−^h^+^ (electron/hole) charge-carrier separation via Lorentz forces in opposite directions and suppress recombination phenomena [27,34,35,36]. Furthermore, the application of an external magnetic field can result in the exertion of Lorentz force to both the photocatalyst as well as the pollutant in opposite directions, achieving proximity and contaminant adsorption on the catalyst surface [37]. In the case of ferromagnetic photocatalysts, an external magnetic field also leads to electron spin alignment in the material’s domains, frequently resulting in negative magnetoresistance, which facilitates charge transfer [36]. Overall, composites with ferromagnetic materials show increased pollutant degradation rates with the increase in the applied external magnetic field strength [38], and, in cases where the resulting alignment of magnetic moments is the same for different components of the composite, facile electron migration through the interface has been reported [39]. Additionally, the manipulation of the photocatalysts’ electron spin polarization states by methods such as doping has also been thought to suppress charge-carrier recombination. In such magnetic semiconductors, flipping the electrons’ spin state can occur via spin–orbit or hyperfine coupling and the recombination between photoexcited e^−^ and h^+^ can be prohibited [36]. Most importantly, the presence of a magnetic material as part of a photocatalytic composite makes it easily recoverable after the end of the photocatalytic process by applying an external magnetic field. The ability to easily retrieve a magnetic photocatalyst also allows for their feasible reuse, as the retrieved material can have its adsorbed contaminants desorbed to render it able for repeated water treatment processes [40].

**Figure 2:**
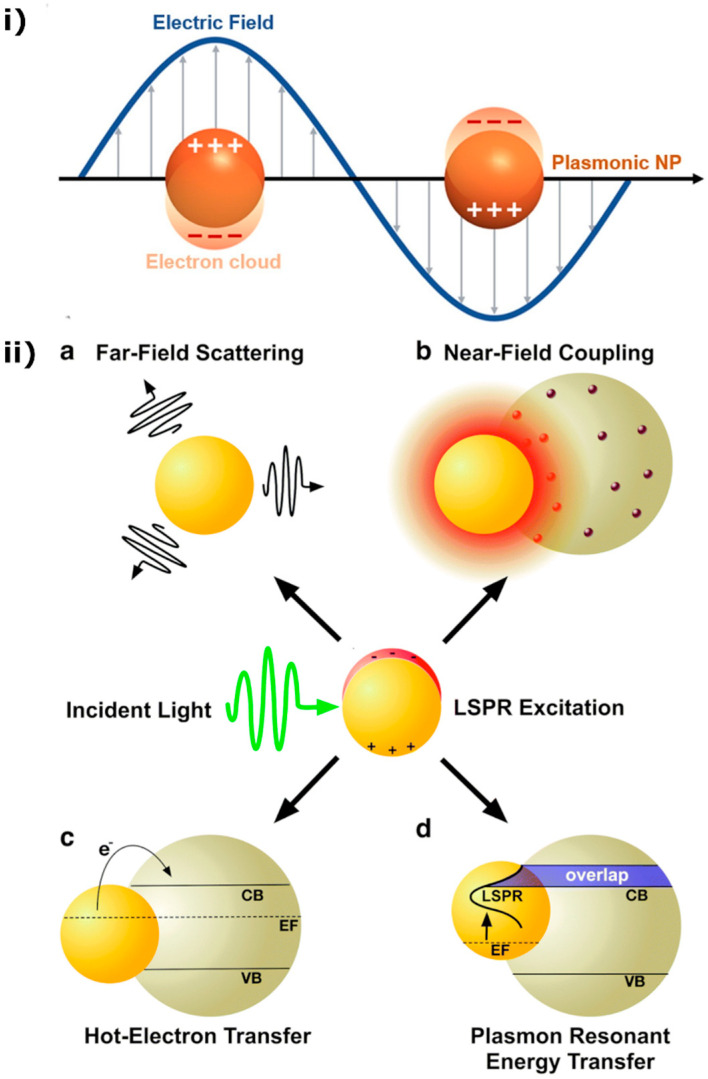
Schematic representations of (**i**) LSPR (Reused with permission [52]. Copyright Elsevier 2017) and (**ii**) Plasmonic enhancement mechanisms (Reproduced from Ref. [43] with permission from the Royal Society of Chemistry).

Lately, the combination of silver with magnetic materials has attracted significant interest. Most of their enhancements brought about by silver nanoparticles are based on their localized surface plasmon resonance (LSPR) effect, caused by their surface electrons’ dipolar oscillation induced by the polarizing incident light’s electric field (Figure 2(iia)). In metal nanoparticles, the electric field of incident light causes displacement of the free electrons from the stationary positive charge (core) and a restoring force that appears, leading to their dipolar oscillation [11]. The term “surface plasmons’’ refers to this surface-localized oscillation of the metals’ free charge. When the incident light frequency matches the natural oscillating frequency of these surface electrons, the LSPR effect is activated with the occurrence of increased light absorption [11,18]. 

The merging of a semiconductor with plasmonic nanoparticles (NPs) is a very effective strategy for enhanced photocatalytic pollutant degradation [41]. Because of the LSPR-induced light absorption enhancement, plasmonic nanoparticle integration can improve a semiconductor’s response to light. In cases of plasmonic NPs with visible light activated-LSPR, the photoactivity of even wide band gap semiconductors, such as TiO_2_, can be extended toward the visible region with their integration [42]. Among the most well-known LSPR-induced enhancement mechanisms in composites are: (a) the light scattering mechanism that lengthens the photons’ effective path [43], (b) a local electric field enhancement on the plasmonic particle surface which results in greater charged-pair production in that area [44], (c) the electron injection mechanism of ‘’hot’’ (excited with high kinetic energy) electrons that can overcome the Schottky barrier (at the semiconductor/metallic nanoparticle interface) and transfer to the semiconductor from the plasmonic NPs [42], and (d) the plasmon-induced resonance energy transfer (PIRET, a dipole–dipole interaction-based non-radiative energy transfer to the semiconductor, which is especially significant when there is an overlap of the semiconductor band edge with the plasmonic absorption band) [43]. The corresponding mechanisms are visualized in Figure 2(iib). Moreover, a direct electron transfer from the plasmonic NPs to the energy states of the pollutant adsorbate is also possible [45]. Finally, besides the plasmonic-based enhancements, photocatalysis can benefit from the storage of excited semiconductor electrons in the Fermi level of metal nanoparticles, shifting the Fermi potential to more negative values, which improves charge separation. These mechanisms, along with the presence of the Schottky barrier aiding electron/hole separation [46], lead to enhanced photocatalytic activity [18]. Silver nanoparticles, in particular, have an especially intense LSPR effect [42] and are considered to be the noble metal-based nanoparticles with the lowest cost [11]. The frequency of their surface plasmon resonance can be modified through their morphology, thereby tuning their optical response [18]. Thus, visible-light-induced photocatalytic enhancements become possible in silver-integrated semiconductors [47]. 

An important factor to consider when designing materials for water treatment is the sensitivity of these materials to the environment. Though silver NPs have been known to be susceptible to oxidation, good chemical stability can be achieved by utilizing modern synthetic methods designed for this purpose. This is usually done by stabilizing agents in colloidal nanoparticle suspensions. Such agents used in photocatalytic applications are surfactants, silica, polymers, and metal shells, summarized in a relatively recent analytical review [48]. More sophisticated approaches are followed in order to obtain extra stable nanoparticles such as the use of a protective ligand shell of p-mercaptobenzoic acid in a notable Ag NP synthetic process, which results in the formation of a closed-shell superatom with 18 de-localized electrons accompanied by the opening of a stabilizing energy gap [49]. It is important to note that additional components, such as intermediate layers, in SC/silver composites can affect the plasmonic properties of the material and conscious selection and tailoring is required. A thinner interlayer, for example, is known to cause a red shift in the required SPR wavelength [50]. Often, there has to be a compromise between the maintenance of nanoparticle stability and the achievement of efficient plasmonic properties, since the presence of protective layers affects the vicinity of the plasmonic NP toward the SC surface and toward the pollutant adsorbate [48]. For Ag NP, thin protective layers in the subnanometer range are preferred in photocatalytic applications [51].

**Figure 3:**
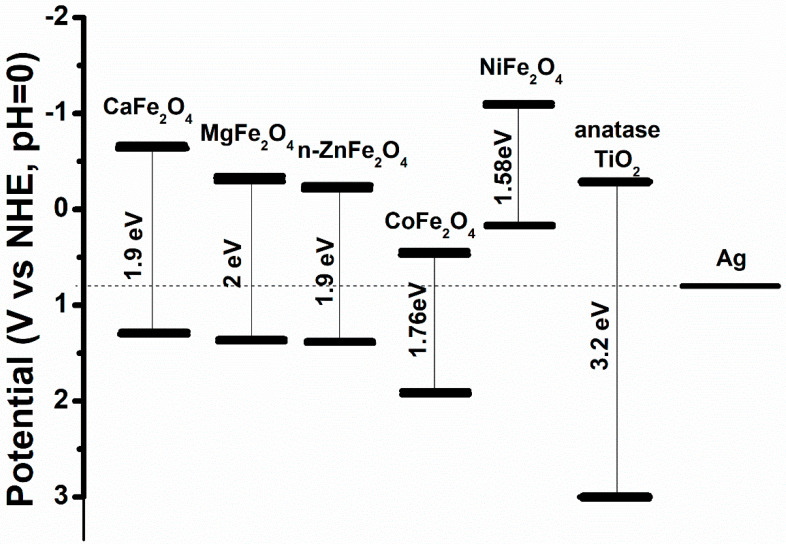
Energy diagram for common spinel ferrites, anatase ΤιO_2_ and silver. The diagram was constructed using the referenced bibliographic works: for Ag [57] and anatase TiO_2_ (reconstruction with permission [58]. Copyright John Wiley & Sons 2010) and for spinel ferrites (reconstruction with permission [54]. Copyright Elsevier 2021).

Another important issue is that, while nanosilver is known to be an excellent antibacterial agent, it has inherent toxicity [53] and, after its function during water treatment is completed, effective separation is needed. For this purpose, magnetic materials are often suggested as base materials for Ag-composites, as the removal of the composites can occur easily with an applied magnetic field [13,14]. In Figure 3, the energy diagram of TiO_2_ and several well-known spinel ferrites [54,55,56] is presented. The position of the energy levels of the semiconductor relative to the silver redox potential defines the number and significance of active photocatalytic enhancement mechanisms, making the choice of the magnetic component very important and often requiring additional SC components for proper energy-level engineering (e.g., ternary composites). 

Another area in which the magnetic substrate can manipulate the integrated plasmonic nanostructures is in their orientational control, thereby allowing for the tuning of the LSPR peak intensity [59]. Plasmonic–magnetic nanocomposites that are responsive to magnetic forces offer a remote and reversible way to control anisotropically shaped plasmonic nanostructures (e.g., nanorods) under external magnetic fields. For example, the selective orientation of plasmonic nanorods parallel to light polarization activates longitudinal LSPR modes with enhanced LSPR peak intensity [60]. 

In general, silver integration is a popular enhancement method for photocatalysts, able to target a variety of pollutants. In a recent work, Ibrahim et al. (2022) observed significantly enhanced photocatalytic pollutant removal efficiency after silver integration for their best TiO_2_/g-C_3_N_4_/Ag sample in both oxidation (azo-dyes/pharmaceuticals) and reduction (Cr^6+^ and 4-nitrophenol) processes [61]. Composites with magnetic materials and silver have been proven to be especially efficient in the treatment of heavy metal pollutants, such as Cr^6+^ [20] or organics such as methylene blue [10,62,63], rhodamine B [10,46,64,65], malachite green [63,66] and phenol [63,67], along with the photocatalytic neutralization of bacteria such as Escherichia coli [63,68] and Micrococcus luteus [63]. Improvements in photocatalytic pollutant removal efficiency arising from silver integration onto magnetic materials are also evident in the case of pharmaceutical pollutants such as tetracycline [69] and sulfanilamide [70]. The silver addition has been proven to enhance the photocatalytic degradation and antibacterial action, not only under UV but under visible illumination as well [10,20,46,62,66,68], even when the base materials are not especially effective under these conditions [20]. In summary, the combined attributes of magnetic materials and silver lead to significantly enhanced composites with usage flexibility.

## 3. Recent Developments in Ag/Magnetic Materials

### 3.1. Synthesis Methods of Magnetic Materials

There are various techniques for the synthesis of magnetic materials: co-precipitation of Fe ions in alkaline solutions [71,72], thermal decomposition of iron precursors in organic solutions [73,74,75], hydrothermal [76,77], solvothermal [78,79], combustion [80], sol–gel auto combustion [81] and microemulsion methods [82] are some of the techniques that have been reported in literature during the last decade (Table 1). In general, a good synthetic process for nanomaterials results in reproducible, monodispersed nanoparticles with controllable characteristics depending on the desired application. For photocatalytic applications, a good synthesis method must allow a degree of tuning for the particles’ shape, size and surface properties, as these parameters directly impact the photocatalytic performance. The nature of its surface is especially important, as it affects properties such as pollutant adsorption (high surface area leads to more sites for pollutant adsorption and the surface charge affects catalyst/pollutant affinity). Furthermore, photocatalysis benefits from nanoparticles with high surface to volume ratios while the prevention of agglomeration into particle clusters is a necessity.

#### 3.1.1. Co-Precipitation Method

**Figure 4:**
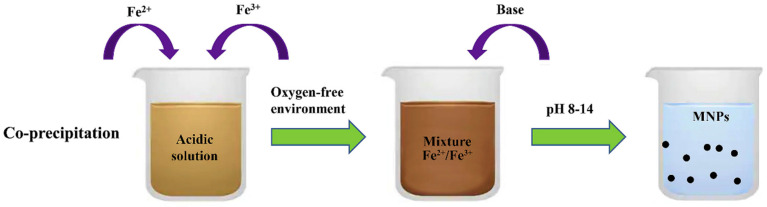
Schematic illustration of co-precipitation synthesis method. (Reused with permission [85]. Copyright Elsevier 2020).

Several authors have reported the synthesis of iron oxide and ferrites NPs by the co-precipitation method [83,84]. The most conventional method used to synthesize either Fe_3_O_4_ or γ-Fe_2_O_3_ is through co-precipitation of ferric (Fe^3+^) and ferrous (Fe^2+^) ions in a 1:2 molar ratio in highly basic solutions at room temperature or at an elevated temperature. The desired pH is created by the addition of basic solutions such as sodium hydroxide solution (NaOH) or ammonium hydroxide solution (NH_4_OH). The characteristics of the magnetic nanoparticles such as size, shape and composition differ and depend on the type of salts used (e.g., chlorides, sulfates, nitrates), Fe^2+^/Fe^3+^ ratio, reaction temperature, types of stabilizing agent, pH value, ionic strength of the reaction media and other reaction parameters (e.g., stirring rate, dropping speed of basic solution). The schematic illustration of the co-precipitation synthesis method is presented in Figure 4.

Furthermore, it should be noted that Fe_3_O_4_ is sensitive to oxygen and may be oxidized to a Fe(OH)_3_ or α-Fe_2_O_3_ phase in the presence of air. In order to avoid this oxidation, the synthesis of Fe_3_O_4_ NPs must be done in anaerobic conditions. However, taking advantage of the easily oxidized Fe_3_O_4_ NPs, Fe_2_O_3_ NPs can be easily prepared by oxidation or anneal treatment under oxygen atmosphere that exhibits chemical stability in alkaline or acidic environment. The co-precipitation method is environmentally benign since only deionized water is used for salt dissolution, in contrast with other synthesis techniques during which toxic organic solvents are used during the synthesis [86]. The drawbacks of this method are the wide size distribution of the synthesized nanoparticles that may need secondary size selection and the high pH value required of the reaction mixture, which has to be adjusted in both the synthesis and purification steps. The generated wastewaters have high basic pH values that demand subsequent treatments in order to avoid environmental harm [27,87]. 

#### 3.1.2. Thermal Decomposition Method

Thermal decomposition of organometallic or coordinated iron precursors in high boiling organic solvents in the presence of various stabilizing surfactants has become an established technique to achieve uniform and monodisperse magnetic nanocrystals. The iron precursors participating in this synthesis strategy can be acetylacetonates, acetates, oleates, carbonyl, oxalates, ferrocene or Fe-urea complex, while benzyl ether or octadecene are usually employed as high-boiling solvents. As for the stabilizers, oleic acid, alcohol, 1-octadecene, 1-tetradecene and oleylamine, are often utilized. Technically, thermal decomposition techniques can be categorized into hot-injection approaches, where the precursors are inserted into a hot reaction mixture, and conventional reaction strategies where a reaction mixture is prepared at room temperature and heated afterward [73,88]. The organic thermal decomposition method has been confirmed to be a promising synthesis technique for synthesizing high quality, monodispersed and highly crystallized magnetic NPs, mostly due to the high temperature of the reactions. Nevertheless, the method possesses certain disadvantages such as high reaction temperature requirement, complicated procedures, usage of numerous reagents, probable emission of toxic gases (such as CO) and use of high cost and toxic reagents. The particles obtained are usually insoluble in water or only soluble in certain non-polar solvents due to the non-polar characteristics of the initial oleate precursor ligand shell. Consequently, additives such as polymers or long-chained water-soluble hydrocarbons must be used to render them appropriate for environmental applications. Therefore, future research in this synthesis method should focus on the preparation of water-soluble magnetic NPs directly with the use of a more limited number of reagents [27,89].

#### 3.1.3. Combustion Method 

The combustion method was introduced to accelerate the synthesis of complex materials. This method is characterized by its simpler process, high energy efficiency, cost effectiveness and rapid nature [90]. Its energy efficiency arises from the fact that high reaction temperatures are self-sustained by the exothermic nature of this method [91]. For this reason, a solution combustion method has been commonly utilized to develop simple and mixed-metal oxides. Organic compounds such as glycine, urea, citric acid, alanine and carbohydrazide are mixed directly with metal nitrates to improve the efficiency of the combustion synthesis technique. The metal nitrates function in a dual way, both as oxidants and as cation sources, while the organic compounds act as the fuel. Normally, conventional heating is used in the processes listed above; however, recently, microwave irradiation heating is becoming popular. It offers a clean, inexpensive and convenient heating method that often results in higher yields, and the reaction process can be completed within a few minutes [92]. Favorably, stoichiometry and crystallite size are easily controlled in the combustion method. The produced material characteristics such as crystallite size, surface area, size distribution and size of agglomeration depend mainly on enthalpy or flame temperature generated during combustion, which is reliant on the nature of the fuel and fuel/oxidizer ratio [93]. 

#### 3.1.4. Sol–Gel Auto Combustion Method

**Figure 5:**
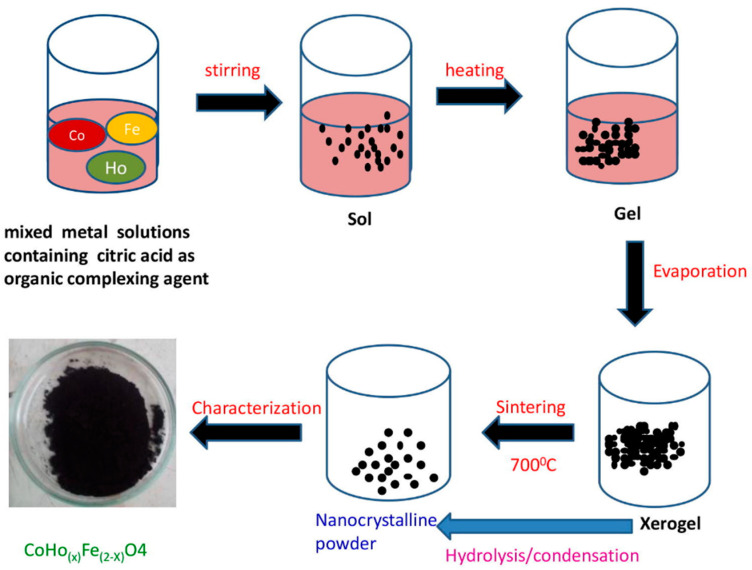
Pictorial representation of sol–gel auto combustion route. (Reused with permission [94]. Copyright Elsevier 2018).

In sol–gel auto combustion synthesis [94,95], a sol is prepared by polymerization or hydrolysis reactions through addition of appropriate reagents in the precursor solution. Then, the gelation process is conducted through polymer addition or sol condensation to gel. Usually, auto combustion is held in order for the magnetic material to be formed. The temperature and time of the self-ignition depends on the material and anticipated characteristics. The sol–gel method is a useful and attractive technique for the preparation of nanosized particles because of its numerous advantages such as reproducibility, high ratio of surface to volume products, good stoichiometric control and the production of ultrafine particles with a narrow size distribution in a relatively short processing time at lower temperatures [85,96]. Figure 5 shows an example of the sol–gel auto combustion method. A modified sol–gel method known as Pechini method has also been referenced in literature [97]: In brief, iron (II) and copper (II) salts were added in an aqueous citric acid solution. After gelation, a suitable amount of ethylene glycol was added, followed by several calcination steps to synthesize efficient and reusable magnetic CuFe_2_O_4_–Fe_2_O_3_ catalysts. 

#### 3.1.5. Solvothermal and Hydrothermal Processes

A solvothermal synthesis method can be defined as a reaction using an organic solvent such as methanol, ethanol, ethylene glycol or polyol in a closed system at a temperature higher than the boiling point of the solvent [98,99]. In the literature, many surfactants used as capping agents during the solvothermal preparation of monodispersed magnetic NPs have been referenced, such as polyacrylic acid, oleic acid and sodium dodecyl benzene sulfonic. The polyol process is categorized under solvothermal processes, but with the usage of specific solvents called high-boiling polyols such as ethylene glycol, diethylene glycol, tri-ethylene glycol, tetra-ethylene glycol and propylene glycol to reduce metal salts to metal particles. The polyols possess a triple role as a high-boiling solvent, reducing agent and stabilizer to control the particles’ growth and inhibit aggregation. Additionally, polyols in solvothermal process is the simplest and most effective procedure for size and morphology adjustment of the magnetic NPs and the process is easy to scale-up. Its main disadvantage is the high sensitivity to the concentration of water and alkalinity, making it a challenge to control the size and surface properties of the produced magnetic NPs [27]. 

Regarding the hydrothermal route [76], a solvothermal method using water as the dispersion medium instead of organic solvents, one of its main advantages, is the possibility to enhance the dissolution of iron precursors. In the hydrothermal synthesis, organic compounds and polymers are usually used as dispersants and stabilizers. The technique is characterized as more cost-effective, resulting in a high yield of products and excellent particle crystallinity with controllable size and good morphology. As an alternative, hydrothermal synthesis includes various wet-chemical technologies of substance crystallization in a sealed container from the high-temperature aqueous solution (generally in the range from 130 to 250 °C) at high vapor pressure (generally in the range from 0.3 to 4 MPa). For example, ferrites can be prepared via the hydrothermal route at a temperature of ~150 °C, whereas the solid-state method requires a temperature of 800 °C [100]. The principal drawbacks of the solvothermal/hydrothermal methods are the slow kinetics due to the lower temperature used. Microwave or ultrasound irradiations are more effective and appealing methods to develop nanoparticles with controllable size and morphology. Ultrasound–hydrothermal, microwave–hydrothermal, ultrasound–solvothermal or microwave–solvothermal routes are able to accelerate the kinetics of reaction, achieve more homogeneous heating, promote nucleation and produce smaller particles [27]. 

#### 3.1.6. Microemulsion Process

Μicroemulsion systems refer to thermodynamically stable colloidal dispersions of immiscible water and oil phases, which are stabilized by the arrangement of surfactant and co-surfactant molecules at the interface [101]. Microemulsions are characterized by droplets with a hydrodynamic diameter of 5–50 nm that are impulsively created by mechanical stirring. These surfactant-covered water droplets can be considered as nanoreactors for the synthesis of NPs. Μicroemulsion synthesis has been widely used for the synthesis of magnetic NPs. When two water nanodroplets collide, they fuse and interchange reactants. When a target particle approaches a water droplet, its surface can adsorb the surfactants, thus helping prevent excess aggregation between particles. Consequently, the particles obtained are generally very fine and monodispersed. In this system, the aqueous phase may contain metal salts and/or other ingredients, and the “oil” may actually be a complex mixture of different hydrocarbons and olefins. The surfactant molecule lowers the interfacial tension between water and oil, resulting in the formation of a transparent solution. Thus, microemulsions are isotropic and stable solutions containing at least three components, a polar phase (frequently water), a non-polar phase (frequently oil) and a surfactant. The two basic types of microemulsions are direct (oil dispersed in water, o/w) and reversed (water dispersed in oil, w/o), which have all been used to synthesize magnetic NPs with tailored shape and size. Surfactants commonly referenced in the literature for the production of magnetic iron oxide and ferrite NPs are sodium di-2-ethylhexyl sulfosuccinate (AOT), sodium dodecyl sulfate (SDS), cetyltrimethylammonium bromide (CTAB) and polyvinylpyrrolidone (PVP). The characteristics of the synthesized NPs can be controlled by the droplet size, the initial concentration of reactants and the nature of surfactants [89]. Additionally, multiple doped and co-doped ferrites have been synthesized through microemulsion technique [82,102,103]. The microemulsion preparation method provides advantages such as economic, environmentally friendly synthesis and unvarying NPs without the need of any size-selection process. Nevertheless, the main drawbacks of this process are the usage of large amounts of solvent (to synthesize a considerable amount of NPs) and the uncontrollable effects of the remaining surfactants on the properties of the particles. Thus, considerations of scale-up fabrication depend relatively on the amount of magnetic NPs that could be synthesized in a single reaction [27].

### 3.2. Magnetic Silver-Integrated Composite Materials

**Figure 6:**
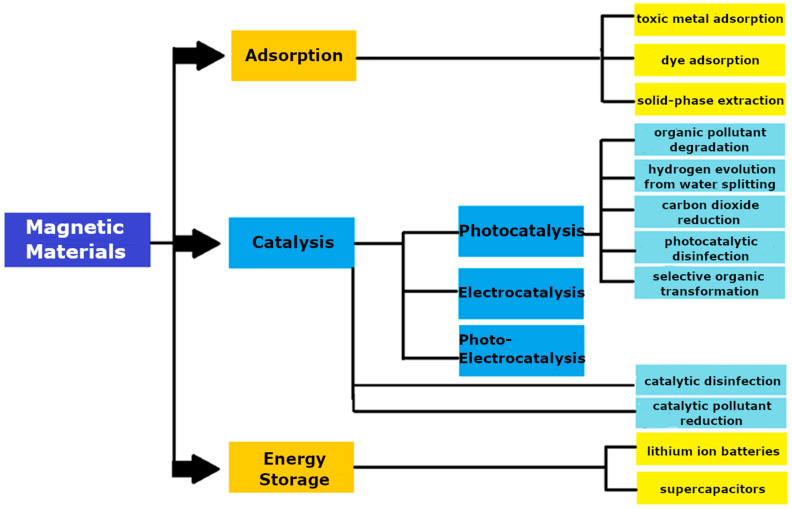
General fields of applications for magnetic materials.

In general, magnetic and hybrid magnetic materials, usually based on iron oxides and ferrites, are commonly used in catalysis for the reduction in pollutants, disinfection, as adsorbing agents, in energy storage field as supercapacitors and in lithium ion batteries [104,105,106,107,108]. Their potential fields of application are summarized in Figure 6. 

In Table 2, recent hybrid magnetic materials containing silver NPs are presented along with their synthesis methods, the particle size(s) and the applications they were tested in.

The most common method for Ag integration is the reduction of silver ions in a solution or gaseous medium at high temperatures [72]. In most cases, the integration of Ag NPs is realized as a separate synthesis step: In a typical process [98,112,113], the base catalyst or catalyst composite is mixed with a silver-containing compound (such as AgNO_3_) in a solution that is usually aqueous. The reduction of silver ions (Ag^+^) can occur through an added reducing agent or via irradiation (photodeposition), leading to Ag NPs (Ag^0^). However, there are cases in the literature where the incorporation of Ag NPs through ion reduction reaction occurs simultaneously during the synthesis of the magnetic material. An example can be found in the work of Khan et al. [62], who synthesized a Ag/rGO/CoFe_2_O_4_ nanocomposite using a one-step hydrothermal technique: amounts of Fe(NO_3_)_3_·9H_2_O, Co(NO_3_)_2_·6H_2_O and AgNO_3_ were added to an aqueous GO solution followed by stirring, pH adjustment and subsequent heating. 

Generally, the use of Ag NPs appears to have two primary drawbacks: their aggregation and the danger of their release into the environment. Thus, combination with magnetic NPs reduces the possibility of aggregation and allows for easy recovery (and reuse) [114]. In general, a small degree of Ag loading (<2 wt%) is preferred [26,115,116,117,118,119,120]. On the other hand, magnetic nanoparticles can be subjected to agglomeration due to the magnetic forces arising between them [20]. For photocatalysts, in general, large surface to volume ratio particles are desired for water treatment applications and there have been several reports highlighting the increased pollutant removal capability of small-sized magnetic nanoparticles [121]. For magnetic particles, specifically, smaller particle size can lead to additional properties such as superparamagnetism [32]. The agglomeration of magnetic particles can limit their potential in many applications and its prevention is often a main concern. Often, a suitable coating material is required to hinder their interaction with the complex matrices and increase their target activity and selectivity. With the use of a proper anchoring agent, formation of a hybrid core–shell material not only prevents the oxidation of ferrite NPs but also stabilizes, adds functionality to the system and further inhibits agglomeration phenomena [72]. Consequently, the coating with an additional material such as silica (SiO_2_) [122], carbon [123] and polymer [124] can protect the magnetic particles from unwanted degradation influenced by the outside environment (possible chemical degradations such as dissolution in acidic media or oxidation of iron oxides under aerobic conditions [27]). Carbon-based materials are used in surface coating of magnetic NPs to enhance their stability, biocompatibility and dispersivity [125]. Silica is the most widely used material for surface modification of magnetic NPs as it provides low agglomeration, enhancement of stability and reduction in cytotoxicity [125,126]. The most common polymers used for the shells of magnetic materials are dextran, chitosan, alginate, polyethylene glycol (PEG), polyvinyl alcohol (PVA), polydopamine (PDA), polysaccharide, polyethylenimine, polyvinylpyrrolidone (PVP), poly acid polyetherimide and polyamidoamine [125]. Furthermore, spacers introduced between the magnetic and silver components (e.g., polyacrylate molecules [127]) during the synthesis of such composites can inhibit magnetic interactions between particles. As mentioned before, the plasmonic properties of SC/silver composites are affected by the presence of intermediate layers and that is the case for magnetic silver-integrated composites as well. Thus, conscious design is needed.

### 3.3. Effects of Silver Integration into Magnetic Photocatalysts

The physicochemical characterization of materials, and especially compounds, is vital for their understanding [20,128,129,130]. In general, for efficient magnetic/silver nanocomposites, a uniform positioning of the silver particles on the surface of the base magnetic material with low agglomeration is optimal [12,20]. 

Regarding optical characteristics, magnetic materials such as the MFe_2_O_4_ family (spinel ferrites) are expected to present significant absorption in the visible range [20,26] and are very popular for photocatalytic purposes, especially when combined with plasmonic nanoparticles [20,26,115,116], whereas the plasmonic nanoparticles themselves enhance visible-light absorption. This is the case for other magnetic materials as well; very recently, it was observed that in core–shell composites comprising a plasmonic core and a magnetic Fe_3_O_4_ coat, silver integration led to the highest absorbed photon flux (compared to plasmonic Au and Al) [131]. Furthermore, plasmonic particle integration is known to result in enhancements in photocurrent generation and transfer [18,20,132,133], which are vital properties for photocatalytic materials. As for the surface properties of photocatalysts, Ag integration into a composite material commonly results in increased surface area [26,115,117,118], which benefits photocatalytic pollutant degradation reactions (materials with a higher specific surface area have more pollutant adsorption sites). Concerning surface chemistry, silver integration influences the surface charge of the final catalyst, which affects the photocatalyst/reactant affinity and its capability for the adsorption, depending on the pH of the specific solution where it is used. A significant point-of-zero charge (PZC) increase is expected after silver integration, which is another reason for the Ag-composite materials’ improved photocatalytic treatment of pollutants such as Cr^6+^. In general, Cr^6+^ ions exist in several anionic forms such as HCrO_4_^−^, CrO_4_^2−^, HCr_2_O_7_^−^ and Cr_2_O_7_^2−^ in aqueous solution with pH value ~2; hence, a photocatalyst with a significantly high PZC value is positively charged and strongly attracts the anionic chromium pollutants, enhancing the photocatalytic reaction [20]. Finally, concerning the matter of silver loading percentage, excess content can inhibit photocatalytic performance by preventing photocatalyst/pollutant contact or by leading to increased recombination sites, among other effects [118,134]; thus, usually, a smaller relative amount of silver (<2%) is chosen for the composite [26,115,116,117,118,119,120].

**Figure 7:**
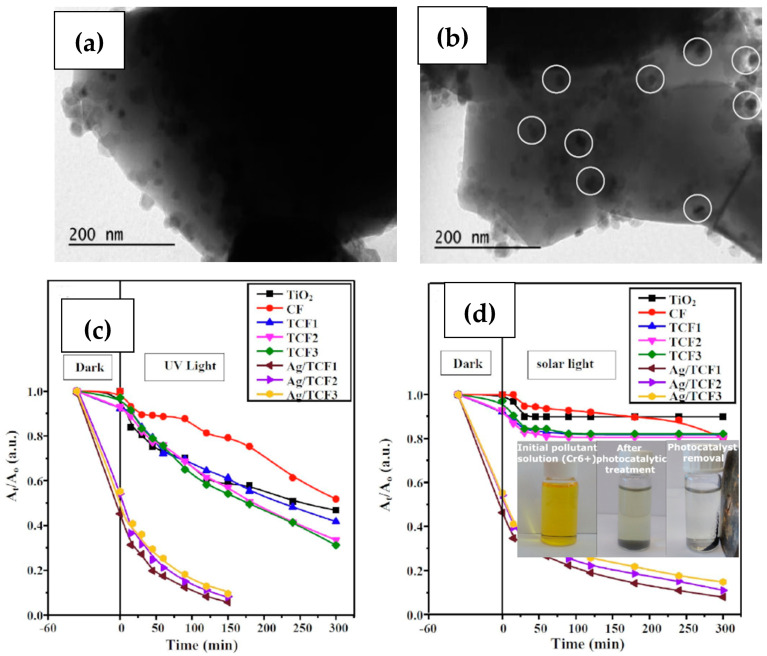
TEM images of a magnetic CoFe_2_O_4_/TiO_2_ (TCF) composite without (**a**) and with (**b**) Ag nanoparticle integration (white circles signify Ag nanoparticles), alongside reduction kinetics of Cr^6+^ under UV (**c**) and artificial solar light (**d**) using the TCF/Ag photocatalysts, and the depiction of the photocatalyst’s magnetic removal after completion (**d**, inset). (Reused with permission [20]. Copyright Elsevier 2019).

Finally, a significant issue for silver-integrated composites is the toxicity of the remnant photocatalyst nanoparticles in the water after their purification activity is completed. This is an even more serious problem for especially small-sized nanoparticles, which are not susceptible to centrifugation or filtration [135]. This is one of the main reasons for the prominent place of magnetic photocatalysts in research and a main advantageous utility in magnetic silver-integrated composites, as an external magnetic field can allow for easy removal of potentially toxic composites from the treated water solution. Thus, materials with a strong response to applied magnetic fields, such as ferrites [136], are advantageous for these applications. However, the formation of composites with the combination of magnetic materials along with non-magnetic components (such as silver nanoparticles) can impact these important magnetic properties. A key intrinsic property for all magnetic nanoparticles is saturation magnetization (Ms), the maximum magnetization possible (during total magnetic dipole alignment) [137,138], as it is a significant design parameter for applications employing ferrimagnetic materials [136]. The value of saturation magnetization can show a decrease with the integration of non-magnetic particles [62], but in most cases, this decrease is not significant enough to prevent the easy removal of composites from the solution through magnetic means [20,135]. Depending on the starting magnetic material and the preparation method, even an increase in magnetic saturation is possible with Ag-integrated composites [139]. Because of these properties, an Ag/magnetic material catalyst can be introduced into a pollutant solution, effectively perform pollutant removal under irradiation and then be easily removed from the “purified’’ solution with means as simple as a magnet (inset, Figure 7d). The same is true for the post-processing of anti-bacterially treated (owing to Ag’s antibacterial nature) pathogenic microorganism (e.g., E. coli) solutions, avoiding the issue of Ag toxicity. 

## 4. Critical Summary of Recent Developments in Magnetic Silver-Integrated Composites for Photocatalytic Water Purification

The advantages of silver integration into magnetic materials for the purposes of enhanced photocatalytic pollutant removal have been examined in a large number of research works in the last years. As for the more prominent magnetic materials utilized in recent works, Fe_3_O_4_ is an especially prevalent semiconductor, often combined with g-C_3_N_4_ in silver-integrated photocatalytic composites, especially for the photodegradation of azo/rhodamine-based dyes [65,140,141,142,143,144,145,146]. Additionally prominent in such works are several types of spinel ferrites (MFe_2_O_4_), based on M = Mg [134], Co [20,147], Ni [148], Zn [70,149,150] and even mixed ferrites such as Ni_0.5_Zn_0.5_Fe_2_O_4_ [151,152].

In Table 3, a bibliographic survey of the recent achievements in the photocatalytic performance of magnetic photocatalysts, with and without silver integration, toward pollutant removal is presented. In a typical process, the catalyst is added to a solution of the target pollutant and the concentration of the pollutant is monitored as a function of the irradiation time. Regarding the photocatalytic performance during aqueous organics degradation, several experimental parameters have significant impact on the photocatalytic efficiency, the most important of which are the initial organics concentration, the photocatalyst’s concentration, the pH and the irradiating light source’s emission spectrum. Regarding irradiation wavelength, photocatalysts are usually more effective under UV-C irradiation and their performance is expected to be superior, e.g., under UV-A compared to visible light, due to the higher energy of lower wavelength photons. This is the case, even with visible light enhancing plasmonic nanoparticles. Concerning pollutant/photocatalyst concentrations, for the same photocatalyst concentration, higher initial pollutant concentrations (mg/L) present increasing difficulty for their treatment due to the excess of organic molecules compared to the available redox species, as well as by blocking the photocatalytic active sites with the byproducts of the photocatalytic reactions. As for the photocatalyst (for a constant initial organic pollutant concentration), there is an optimal concentration above which the solution slurry turbidity (measure of relative clarity in the liquid) limits light transmission and below which the impaired active species formation significantly reduces the photocatalytic efficiency [118]. 

In almost all cases (Table 3), silver integration leads to significant removal efficiency improvement, especially under visible light irradiation. This improvement is evident not only in the cases of organic dye pollutants [63,65,144,146,148,149] and hexavalent chromium [20,146] but also in the case of pharmaceutical pollutants [69,70]. Summarizing the findings of the works on magnetic composites, the base catalyst’s surface area has been reported to increase after silver integration [26,117,118], leading to a greater number of active sites for the photocatalytic reactions, while the resulting point-of-zero charge value increase (representing the surface charge of the material) leads to a greater affinity to negatively charged reactants [20]. However, the majority of the reports regarding photocatalytic performance improvement of a magnetic material after Ag integration attribute it to the improvements in visible light response [20,69,140,144,149] and charge separation [20,63,140,141,144,146]. Regarding the light response enhancements, the silver’s LSPR effect is tuned through the synthesis method to a specific desired frequency [18,156]. When light of that frequency passes through the catalyst, a greatly enhanced light absorption occurs along with the presence of the aforementioned strong electric fields on the plasmonic silver nanoparticles’ surface [18]. An increase in the light absorption capability of a photocatalyst leads to more energy for charged-pair generation, which is further improved by the local electric field enhancement [157], and thus, more active agents become available for photocatalysis reactions. The end result is a significant improvement in the photocatalytic pollutant degradation performance of the catalyst, triggered by light of a specific frequency (in most observed cases, studies focus on the utilization of visible light). Regarding the charge-separation improvement, the interaction between the local electric field on the silver nanoparticles and the base semiconductor leads the generation of electrons and holes to occur in close proximity to the surface of the semiconductor, which, along with the Schottky junction formed in the interface, allow facile charge separation [158], avoiding issues of short hole diffusion lengths and recombination. The improvements in the electrochemical properties of magnetic composites with the integration of silver, can be observed in the produced photocurrent increase and improved charge-transfer properties [20,133]. It is important to note that these plasmonic enhancements in silver-integrated composites lead to impressive results for photocatalytic processes in general, including hydrogen generation and carbon dioxide reduction [159].

**Figure 8:**
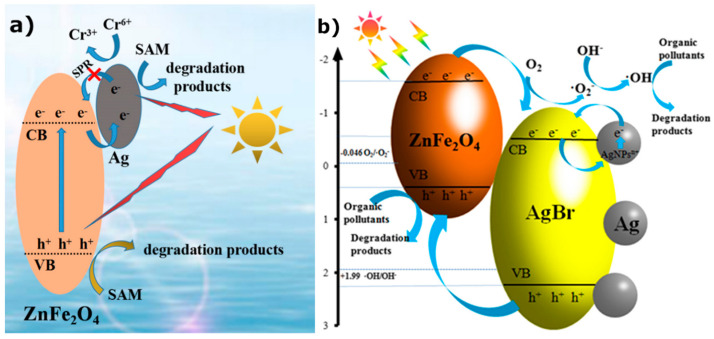
Schematics of photocatalytic processes: (**a**) for the degradation of the antibiotic sulfanilamide (SAM) and the reduction of hexavalent chromium (Cr^6+^) by a magnetic/silver composite (Ag/ZnFe_2_O_4_). (Reused with permission [70]. Copyright Elsevier 2021) (**b**) for the degradation of organic pollutants by a visible-light-activated Ag/AgBr/ZnFe2O4 composite. (Reused with permission [155]. Copyright John Wiley & Sons 2020).

Furthermore, depending on the structure and components of the silver-integrated composite, different mechanisms of charge transfer are possible. The photocatalytic enhancement observed in a Ag/ZnFe_2_O_4_ nanocomposite can be attributed to electron storage in the Fermi energy of the Ag nanoparticles, as shown in Figure 8a [70]. However, in the case of Ag/AgBr/ZnFe_2_O_4_ (Figure 8b) [155], photoexcited electrons from the ferrite as well as plasmon-excited electrons from the Ag nanoparticles are transferred to the CB of AgBr, resulting in efficient generation of photocatalytic active species.

As for the magnetic properties of magnetic silver-integrated composites specifically, the ease of separation and recovery of the composite photocatalyst from the treated solution is often reported [20,63,153,160]. Beyond the magnetic separation capability, there is a great untapped potential regarding the utilization of other magnetic-based capabilities of magnetic silver-integrated composites for photocatalytic water purification applications, though such composites offer an array of useful properties as discussed above. Especially interesting are the effects of tuning the plasmonic characteristics of these composites, such as the function of the remote and reversible orientational control (through the use of a magnetic field) for controlling the LSPR peak intensity of plasmon structures on magnetic substrates, which has been utilized in cancer diagnosis [59]. Another even more fascinating result that has been recently reported in Au-decorated Fe_2_O_3_-TiO_2_ nanotubular structures is the anisotropic magnetic-field-induced tuning of the photocatalytic activity [161]. This effect is attributed to the magnetization of the material and the interaction of the Fe_2_O_3_ magnetic moments with the electron spins of the plasmonic nanoparticles, which results in an increase in the LSPR intensity and improvement of the charge-carrier transfer efficiency. Though it was only observed for Au nanoparticles in this work, this is clear evidence of a synergetic effect of plasmonic and magnetic functionalities in photocatalysis.

## 5. Conclusions and Future Perspectives

A review of recent progress in magnetic photocatalysts with integrated silver for photocatalytic water purification was performed. The combination of magnetic semiconductor materials with plasmonic silver nanoparticles leads to several advantages such as an extended wavelength light response and suppressed recombination. Because of the magnetic component in the composite, an ease of magnetic separation from the treated water solution with the use of an external magnetic field becomes possible, avoiding the issue of silver’s inherent toxicity. Initially, a review of recent preparation methods for magnetic and magnetic silver-integrated materials was performed. Τhe three most conventional synthesis methods of magnetic materials are co-precipitation, solvothermal and sol–gel auto combustion methods, because of their effectiveness and reproducibility. As for silver integration, the most common method is the chemical reduction of silver ions added in a medium along with the starting material (either during starting material synthesis or as a separate step). The prevention of aggregation among magnetic NPs and among silver NPs are two of the most critical issues regarding synthesis. Though the magnetic component aids in aggregation prevention of silver NPs and vice versa, aggregation can be further prevented through suitable incorporation of an additional material as a coating/shell for the magnetic component. Silver integration leads to light absorption and photocurrent generation enhancements and a potential decrease in saturation magnetization is usually not significant enough to prevent the post-reaction facile removal of the magnetic composite from the treated solution. Lastly, the most recent photocatalytic applications in pollutant removal by such composites are presented. The significant performance enhancement of magnetic photocatalysts after Ag integration is evident by the improved pollutant removal efficiency—tested against a variety of pollutants from organic dyes to hexavalent chromium—under UV and visible light, with the enhancement attributed most commonly to the improved visible light response and charge carrier generation/separation/transfer and with the facile magnetic recovery adding flexibility to their usage. 

Regarding perspectives for future research, magnetic composite materials remain a rich source of study for photocatalytic and antibacterial applications, especially when combined with plasmonic nanoparticles. An area of study that requires further research is the impact of magnetic fields on the photocatalytic performance of magnetic silver-integrated composites for pollutant degradation applications. External magnetic fields applied during a photocatalytic process are known to influence the behavior of the active species, and this influence extends to properties such as charge-pair separation, directly affecting the photocatalytic performance. The effect of magnetic properties on the plasmonic characteristics of such composites is especially interesting, an example being the tuning of LSPR intensity through magnetic orientation. Thus, more insight is needed regarding these effects in magnetic/plasmonic composites in order to fully utilize the combined properties of such components. Additionally, the unique facile magnetic retrieval advantage requires further study in magnetic composites with silver. More extensive recyclability studies are needed in order to evaluate the materials’ properties and efficiency in the repeated long-term reuse of magnetic silver composites for photocatalytic pollutant degradation and to establish the way for effective actual water purification systems utilizing robust recycling of photocatalysts for extended utility. 

Furthermore, of great interest are the study of the interlayer in ternary magnetic composites with integrated plasmonic particles and the study of the complicated interactions between components in magnetic multi-component composites, in general. There has been increased research into coating materials for magnetic nanoparticles for prevention of agglomeration and for stability and dispersity enhancement. These studies become more complicated with the addition of plasmonic nanoparticles as part of the composite, as the properties of the intermediate layer between magnetic base material and plasmonic nanoparticles directly affect the attributes of the plasmons, such as their LSPR frequency. Conscious tuning is required for optimal tailoring of the characteristics of the final composite. In conclusion, the promising combination of magnetic and plasmonic properties lead to enhanced performance and flexible usage in photoactivated water purification applications and validates the increased interest in silver-integrated magnetic composites with conscious component tailoring, which is expected to lead to even greater advances in the future.

## Figures and Tables

**Table 1:** Summary of synthesis methods.

Synthesis Method	Advantages	Disadvantages
Co-precipitation	- Simple process - Environmentally benign	- Wide particle size distribution of synthesized NPs - Generated wastewaters with high basic pH
Thermal decomposition	- High quality monodispersed NPs - Highly crystallized NPs	- High reaction temperature requirement - Complicated procedure - Possible emission of toxic gases - Use of high cost and toxic reagents
Combustion	- Simple and rapid process - Energy efficiency - Cost effectiveness - Controllable stoichiometry and crystallite size	- High energy demand
Sol–gel auto combustion	- Reproducibility - Products with high surface to volume ratio - Good stoichiometric control - Narrow size distribution of NPs - Lower temperatures needed compared to combustion method	- Heating requirement
Solvothermal and hydrothermal	- Cost-effectiveness - High yield of products - Excellent particle crystallinity - Controllable size and good morphology	- Slow kinetics due to the lower reaction temperature
Microemulsion	- Very fine and monodispersed NPs - Economic method - Environmentally benign	- Usage of large amounts of solvent - Uncontrollable effects of the remaining surfactants

**Table 2:** Recent magnetic silver-integrated composites synthesized with different methods.

9	Composites: Preparation Method (Component)	Particle Size(S)	Target Application Type (Details)	Ref.
Co-precipitation-based	Ag/Fe_3_O_4_: co-precipitation (Fe_3_O_4_)/ ion reduction (Ag integration–separate step)	≈16.2 nm (magnetic NPs)	Catalysis (methane partial oxidation and formation of formaldehyde)	Navarro et al., 2020 [83]
	Ag/PDA/GO/Fe_3_O_4_: co-precipitation (Fe_3_O_4_)/ solvothermal (GO)/ self-polymerization (PDA)/ ion reduction (Ag integration–separate step)	≈20 nm (Ag NPs)	Catalysis (removal of methylene blue and p-nitrophenol by NaBH_4_)	Upoma et al., 2020 [71]
	Ag/CNT/ Fe_3_O_4_: co-precipitation (CNT/Fe_3_O_4_)/ ion reduction (Ag integration–separate step)	-	Catalysis (removal of o-nitrophenol, p-nitrophenol, 2-methyl-p-nitrophenol, and methyl orange with NaBH_4_) Biomedical (antibacterial activity against Escherichia coli and Bacillus megaterium)	Bhaduri et al., 2018 [105]
	Ag/C-QDs/ Fe_3_O_4_: co-precipitation (Fe_3_O_4_)/ hydrothermal (C-QDs)/ ion reduction (Ag integration–separate step)	≈42 nm (magnetic NPs)	Catalysis (removal of crystal violet and p-nitroaniline in the presence of NaBH_4_)	Guo et al., 2017 [84]
	Ag/PE/MnFe_2_O_4_: co-precipitation (MnFe_2_O_4_)/ ion reduction (Ag integration-separate step)	≈100 nm (magnetic NPs)	Catalysis (removal of RhB, MO, CR, MR, AY and 4-NP in the presence of NaBH_4_)	Gürbüz et al., 2021 [72]
Hydrothermal-based	Ag/C/CoFe_2_O_4_: hydrothermal (Fe_3_O_4_)/ calcination (C)/ ion reduction (Ag integration–separate step)	≈50 nm (magnetic NPs)	Adsorption (adsorption of penicillin and ciprofloxacin) Photocatalysis (photocatalysis of azo-dyes)	Bodaghi et al., 2020 [76]
	Ag/rGO/CoFe_2_O_4_: hydrothermal	≈35–46 nm (magnetic NPs)	Electrochemical	Khan et al., 2020 [62]
Solvothermal-based	Ag/PTA/Fe_3_O_4_: solvothermal (Fe_3_O_4_)/self- polymerization (PTA)/ ion reduction (Ag integration–separate step)	≈250 nm (magnetic NPs)	Biomedical (antibacterial activity against Escherichia coli and Staphylococcus aureus bacteria)	Wang et al., 2018 [109]
	Ag/Fe_3_O_4_: solvothermal (Fe_3_O_4_)/ ion reduction (Ag integration–separate step)	≈217 nm (magnetic NPs) ≈23–41 nm (Ag NPs)	Adsorption and catalysis (Hg^2+^ adsorption and reduction)	Inglezakis et al., 2020 [98]
	Ag/PDA/Fe_3_O: solvothermal (Fe_3_O_4_)/ self-polymerization (PDA)/ ion reduction (Ag integration–separate step)	≈420 nm (magnetic NPs) ≈25 nm (Ag NPs)	Biomedical (antibacterial activities against Escherichia coli and Staphylococcus aureus)	Qin et al., 2017 [78]
Combustion and sol–gel auto combustion-based	Ag/MnFe_2_O_4_: sol–gel auto combustion	≈40–50 nm (magnetic NPs)	Biomedical (antibacterial activity toward Escherichia coli)	Ning et al., 2020 [95]
	Ag/CoFe_2_O_4_: sol–gel auto combustion	≈32–58 nm (magnetic NPs)	-	Routray et al., 2020 [110]
	Ag/MgFe_2_O_4_: combustion (MgFe_2_O_4_)/ ion reduction through combustion (Ag integration–separate step)	≈100 nm (magnetic NPs) ≈20–90 nm (Ag NPs)	Biomedical (antibacterial activity)	Lagashetty et al., 2019 [80]
Thermal decomposition-based	Ag/NiFe_2_O_4_: thermal decomposition (NiFe_2_O_4_)/ ion reduction (Ag integration–separate step)	≈35 nm (magnetic NPs)	Biomedical (anti-bacterial and anti-fungi activity toward Bacillus subtilis and Pseudomonas syringae bacteria and Alternaria solani and Fusarium oxysporum, respectively) Catalysis (epoxidation of alkenes)	Golkhatmi et al., 2017 [73]
Not mentioned	Ag/CD-MA/Fe_3_O_4_	≈50 nm (magnetic NPs)	Catalysis (removal of nitroaromatics and organic dyes)	Nariya et al., 2019 [111]

**Table 3:** Photocatalytic pollutant removal performance by magnetic photocatalysts with and without Ag. Pollutants include hexavalent chromium (Cr^6+^), dyes such as methylene blue (MB), congo red (CR), methyl orange (MO), malachite green (MG), and rhodamine B (RhB), and pharmaceuticals such as tetracycline (TC), carbamazepine (CBZ), metronidazole (MZ), sulfanilamide (SAM), gemfibrozil (GEM) and tamoxifen (TAM).

Photocatalyst (Concentration in Pollutant Solution, mg/mL)		Pollutant (Initial Concentration, mg/L)	Irradiation Type	Removal Efficiency (%)	Time (min)	Ref.
TiO_2_/CoFe_2_O_4_	(0.1)	Cr^6+^ (5)	UV/Vis	58.3/18.4	150/300	Ibrahim et al., 2020 [20]
Ag/TiO_2_/CoFe_2_O_4_				95.1/92.1	150/300	
Ag/CoFe_2_O_4_/PANi	(0.05)	MB	Sunlight	~80	180	Mosali et al., 2017 [147]
ZnFe_2_O_4_/ZnO	(1)	MO (10)	Vis	63.4	420	Su et al., 2018 [149]
ZnFe_2_O_4_/ZnO/Ag				84		
Bi_12_O_17_Cl_2_/AgFeO_2_	(0.5)	TC (40)	Vis	77.3	60	Guo et al., 2021 [69]
Bi_12_O_17_Cl_2_/Ag/AgFeO_2_				94.1		
BiFeO_3_	(1)	MG (10)	Vis	~70	240	Jaffari et al., 2019 [63]
Ag/BiFeO_3_				85.5		
Ag_3_PO_4_/Ag/NiFe_2_O_4_	(0.4)	MB (20)	Vis	~99	60	Dong et al., 2018 [153]
Ag/Fe_3_O_4_/ZnO	-	MB	UV	99	120	Tju et al., 2018 [141]
Ag/AgBr/ZnFe_2_O_4_	(1)	CBZ (10)	Vis	22.7	240	Yentur et al., 2020 [150]
AgBr/g-C_3_N_4_/Fe_3_O_4_	(0.4)	RhB (20)	Vis	76	150	Zhang et al., 2021 [65]
Ag@AgBr/g-C_3_N_4_/Fe_3_O_4_				96		
MgFe_2_O_4_/ZnO	(1)	CR (25)	Vis	88	60	Nasab et al., 2020 [134]
MgFe_2_O_4_/ZnO/Ag				82		
g-C_3_N_4_/Fe_3_O_4_/Ag/Ag_2_SO_3_	(0.4)	RhB (12)	Vis	99	270	Akhundi et al., 2017 [140]
Zn_0.5_Ca_0.5_Fe_2_O_4_/Ag	(2)	RhB (40)	Vis	total	120	Fernandes et al., 2021 [151]
Fe_3_O_4_@TiO_2_@PDA/SiW_11_V	(1)	MO (15)	Vis	29	120	Wu et al., 2021 [146]
Fe_3_O_4_@TiO_2_@PDA/SiW_11_V-Ag				total		
Fe_3_O_4_@TiO_2_@PDA/SiW_11_V	(1)	Cr^6+^ (500)		36.5		
Fe_3_O_4_@TiO_2_@PDA/SiW_11_V-Ag				91.3		
Ag/Ni_0.5_Zn_0.5_Fe_2_O_4_	-	MZ (50)	UV	99.9	360	Mustafa, 2021 [152]
ZnO/Fe_3_O_4_	(1)	MB (10)	Simulated Sunlight	63.48	240	Zhang et al., 2021 [144]
Ag/ZnO/Fe_3_O_4_				97.31		
NiFe_2_O_4_-TiO_2_/rGO	(0.15)	MB (10)	Vis	~60	150	Bourzami et al., 2021 [148]
NiFe_2_O_4_-TiO_2_/rGO/Ag				~75		
Ag/ZnFe_2_O_4_	(0.4)	Cr^6+^ (20)	Vis	82.7	120	Liu et al., 2021 [70]
ZnFe_2_O_4_		SAM (20)		48.4		
Ag/ZnFe_2_O_4_				98.4		
Ag-CuFe_2_O_4_@WO_3_	(0.2)	GEM (5)	UV	81	150	Sayadi et al., 2021 [154]
		TAM (5)		83		
Ag/AgBr/ZnFe_2_O_4_	(1)	MO (10)	Vis	96	120	Li et al., 2020 [155]
